# Supplementary material for: MetaQTL: a package of new computational methods for the meta-analysis of QTL mapping experiments
Source: BMC Bioinformatics. 2007 Feb 8;8:49. doi: 10.1186/1471-2105-8-49 (PMC1808479; doi:10.1186/1471-2105-8-49)
Supplement: Additional File 7 — MetaQTL Package : jar file and tutorial. This Zip archive contains both the MetaQTL JAR file and the files of the tutorial. [file 1471-2105-8-49-S7.zip › org.inra.metaqtl/doc/org/metaqtl/Qtl.html]

Qtl


|  |  |  |
| --- | --- | --- |
| **Overview** \| **Package** \| **Class** \| **Use** \| **Tree** \| **Deprecated** \| **Index** \| **Help** | |  |
| **PREV CLASS**   **NEXT CLASS** | **FRAMES**    **NO FRAMES**     **All Classes** |  |
| SUMMARY: NESTED \| FIELD \| CONSTR \| METHOD | DETAIL: FIELD \| CONSTR \| METHOD |  |


---


## org.metaqtl Class Qtl

```
java.lang.Object
  org.metaqtl.Qtl
```

---

``` public class Qtl extends java.lang.Object ```

A QTL (Quantitative Trait Loci)

---

| **Nested Class Summary** | |
| --- | --- |
| `class` | `Qtl.QTLCross`             This class stores the informations on the cross design used to detect the QTL. |
| `class` | `Qtl.QTLProj`             This class stores the QTL projection properties. |
| `class` | `Qtl.QTLStats`             This class stores the usual statistics for the QTL. |
| `class` | `Qtl.QTLSupport`             This class stores information on the QTL Confidence Interval |
| `class` | `Qtl.QTLTrait`             This class stores the trait description of a QTL |


| **Field Summary** | |
| --- | --- |
| `double` | `c1`             The recombination rate between the QTL position and the left flanking marker. |
| `double` | `c2`             The recombination rate between the QTL position and the right flanking marker. |
| `Qtl.QTLSupport` | `ci`             The QTL confidence interval. |
| `Qtl.QTLCross` | `cross`             The QTL cross design. |
| `int` | `id`             The id of the Qtl |
| `int` | `m1`             The indice of the left flanking marker. |
| `int` | `m2`             The indice of the right flanking marker. |
| `java.lang.String` | `name`             The name of the QTL. |
| `double` | `position`             The QTL most probable location on the chromosome. |
| `Qtl.QTLProj` | `proj`             The QTL projection properties. |
| `double` | `sd_position`             The standard deviation of the QTL position. |
| `Qtl.QTLStats` | `stats`             The QTL statistics |
| `Qtl.QTLTrait` | `trait`             The QTL trait data. |


**Constructor Summary**

`Qtl()`


| **Method Summary** | |
| --- | --- |
| `Qtl` | `getCopy()`             Returns a copy of the QTL. |
| `java.lang.String` | `getName()`             Returns the name of the QTL. |
| `static double` | `getQTLSD(double rsquare, Qtl.QTLCross cross, int mappingUnit)`             Depending on the r-square parameter and the cross design this method returns the standard deviation of the QTL position for the given mapping context. |
| `static double` | `getQTLSD(Qtl.QTLSupport ci, int mappingFunction, int mappingUnit)`             Depending on the CI type this methods returns the the standard deviation of the QTL position using the given mapping context. |
| `boolean` | `isIgnore()`             This methods returns true if the QTL must be ignored. |
| `void` | `setIgnore(boolean ignore)`             Set the status of the QTL. |

**Methods inherited from class java.lang.Object**

`clone, equals, finalize, getClass, hashCode, notify, notifyAll, toString, wait, wait, wait`

**Field Detail**

### id

```
public int id
```

:   The id of the Qtl

---


### name

```
public java.lang.String name
```

:   The name of the QTL.

---


### m1

```
public int m1
```

:   The indice of the left flanking marker.

---


### m2

```
public int m2
```

:   The indice of the right flanking marker.

---


### position

```
public double position
```

:   The QTL most probable location on the chromosome.

---


### sd\_position

```
public double sd_position
```

:   The standard deviation of the QTL position.

---


### c1

```
public double c1
```

:   The recombination rate between the QTL position and
    the left flanking marker.

---


### c2

```
public double c2
```

:   The recombination rate between the QTL position and
    the right flanking marker.

---


### trait

```
public Qtl.QTLTrait trait
```

:   The QTL trait data.

---


### stats

```
public Qtl.QTLStats stats
```

:   The QTL statistics

---


### ci

```
public Qtl.QTLSupport ci
```

:   The QTL confidence interval.

---


### cross

```
public Qtl.QTLCross cross
```

:   The QTL cross design.

---


### proj

```
public Qtl.QTLProj proj
```

:   The QTL projection properties.


**Constructor Detail**

### Qtl

```
public Qtl()
```


**Method Detail**

### getQTLSD

```
public static double getQTLSD(Qtl.QTLSupport ci,
                              int mappingFunction,
                              int mappingUnit)
```

:   Depending on the CI type this methods returns the
    the standard deviation of the QTL position using the given
    mapping context.

    :   **Parameters:**: `ci` - the confidence interval of the QTL.: `mappingFunction` - the mapping function.: `mappingUnit` - the mapping unit. **Returns:**: the standard deviation of the QTL position.

---


### getQTLSD

```
public static double getQTLSD(double rsquare,
                              Qtl.QTLCross cross,
                              int mappingUnit)
```

:   Depending on the r-square parameter and the cross design
    this method returns the standard deviation of the QTL position
    for the given mapping context.
    This method uses the formula introduced in Darvasi A. and Soller M.,
    A Simple Method to Calculate Resolving Power and Confidence Interval
    of QTL Map Location, 1997, Behavior Genetics 27,2:125-132.
    For the moment the cross type is not taken into account.

    :   **Parameters:**: `rsquare` - **Returns:**

---


### isIgnore

```
public boolean isIgnore()
```

:   This methods returns true if the QTL must be ignored.

    :   **Returns:**: true if the QTL must be ignored.

---


### setIgnore

```
public void setIgnore(boolean ignore)
```

:   Set the status of the QTL.

    :   **Parameters:**: `ignore` - the status of the QTL.

---


### getCopy

```
public Qtl getCopy()
```

:   Returns a copy of the QTL.

    :   **Returns:**: a copy of the QTL.

---


### getName

```
public java.lang.String getName()
```

:   Returns the name of the QTL.

    :   **Returns:**: the name of the QTL.


---


|  |  |  |
| --- | --- | --- |
| **Overview** \| **Package** \| **Class** \| **Use** \| **Tree** \| **Deprecated** \| **Index** \| **Help** | |  |
| **PREV CLASS**   **NEXT CLASS** | **FRAMES**    **NO FRAMES**     **All Classes** |  |
| SUMMARY: NESTED \| FIELD \| CONSTR \| METHOD | DETAIL: FIELD \| CONSTR \| METHOD |  |


---
